# Pharmacotherapy of Behavioral and Psychological Symptoms of Dementia: State of the Art and Future Progress

**DOI:** 10.3389/fphar.2020.01168

**Published:** 2020-07-31

**Authors:** Radoslaw Magierski, Tomasz Sobow, Emilia Schwertner, Dorota Religa

**Affiliations:** ^1^ Department of Old Age Psychiatry and Psychotic Disorders, Medical University of Lodz, Lodz, Poland; ^2^ Dialog Therapy Centre, Warsaw & Institute of Psychology, University of Lodz, Lodz, Poland; ^3^ Center for Alzheimer Research, Division of Clinical Geriatrics, Department of Neurobiology, Care Sciences and Society, Karolinska Institutet, Huddinge, Sweden; ^4^ Tema Aging, Karolinska University Hospital, Stockholm, Sweden

**Keywords:** behavioral and psychological symptoms of dementia, neuropsychiatric symptoms, antipsychotics, antidepressants, non-pharmacological interventions, clinical trial

## Abstract

The core symptoms of different dementia subtypes are the behavioral and psychological symptoms of dementia (BPSD) and its neuropsychiatric symptoms (NPS). BPSD symptoms may occur at any stage in the case of dementia due to Alzheimer’s disease (AD), whereas they tend to occur early on in the case of its behavioral variant frontotemporal dementia or dementia with Lewy bodies and are essential for diagnosis. BPSD treatment consists of non-pharmacological as well as pharmacological interventions, with non-pharmacological interactions being suggested as first-line treatment. Agitation, psychotic features, apathy, depression, and anxiety may not respond to acetylcholinesterase inhibitors or memantine in AD cases; therefore, antipsychotics, antidepressants, sedative drugs or anxiolytics, and antiepileptic drugs are typically prescribed. However, such management of BPSD can be complicated by hypersensitivity to antipsychotic drugs, as observed in DLB, and a lack of effective pro-cognitive treatment in the case of frontotemporal dementia. The present paper reviews current knowledge of the management of BPSD and its limitations and discusses on-going clinical trials and future therapeutic options.

## Introduction

The commonly-observed core symptoms of dementia are classified as behavioral and psychological symptoms of dementia (BPSD) or as neuropsychiatric symptoms (NPS) (Zhao et al., 2016). NPS are mainly associated with Alzheimer’s dementia (AD), but they can occur in various types of dementia, as well as in mild cognitive impairment (MCI) (Siafarikas et al., 2018). NPS have been recognized as a risk factor of dementia among individuals with MCI, especially when co-occuring with affective and psychotic symptoms (Liew, 2019). Moreover, cases characterized by behavioral changes and psychiatric symptoms, but without cognitive impairment in later life, have been described as mild behavioral impairment (Taragano et al., 2009); this has been recognized as an at-risk state for cognitive decline and dementia, biomarker of cognitive decline, or even a potential manifestation of prodromal dementia (Ismail et al., 2018; Taragano et al., 2018; Creese et al., 2019). While NPSs impair the quality of life of both patient and caregiver, they seem to exert the strongest impact on the latter (Feast et al., 2016; Terum et al., 2017) and may influence the decision regarding nursing home placement (Porter et al., 2016; Vandepitte et al., 2018). In addition, together with the severity of dementia, the presence of NPS contributes to increasing care costs (Rattinger et al., 2015; Wübker et al., 2015; Rattinger et al., 2019).

NPS have a high prevalence index, and different patterns of symptoms are observed depending on the course of the illness, biological factors, diagnosis, age of onset, dementia severity, and place of residence of patients (Lyketsos et al., 2000; Tractenberg et al., 2003; Ryu et al., 2005; Ballard, 2007; Peters et al., 2012; van der Linde et al., 2012; Borsje et al., 2015; Mulders et al., 2016; van der Linde et al., 2016; Bauhuis et al., 2018; Rozum et al., 2019). A good example is AD, in which NPS are extremely common: apathy is the most frequent symptom, followed by depression, aggression, anxiety, and sleep disorder (Zhao et al., 2016). Other symptoms, such as irritability, appetite disorder, aberrant motor behavior, delusion, disinhibition, and hallucination are less common, with their prevalence ranging from 36 to 16% of AD cases. Therefore, it is necessary to implement effective strategies against NPS and their unavoidable serious consequences.

The aim of the paper is to review current knowledge, limitations, and practices regarding the management of BPSD and to discuss on-going clinical trials and future therapeutic options.

## Review of Current Knowledge on BPSD Management

### Non-Pharmacological Strategies

A number of non-pharmacological techniques are used in medicine, specifically in the late-life population, a number of which have been found to be effective at treating neuropsychiatric symptoms (Gitlin et al., 2009; Abraha et al., 2017); however, the quality of evidence for such interventions is low (Wang G. et al., 2018), and there is limited evidence for recommending their use in general (Cabrera et al., 2015). As recent papers on non-pharmacological interventions have higher reporting quality than older ones, it is likely that our knowledge on the role of non-pharmacological interventions will be steadily improved as more studies are performed (Horr et al., 2015).

Non-pharmacological approaches comprise various types of intervention: sensory stimulation (acupressure, aromatherapy, massage, touch therapy, light therapy, garden activities, music and dance therapy, and snoezelen multisensory stimulation therapy), cognitive and emotion-oriented approaches (cognitive stimulation, reminiscence therapy, validation therapy, and simulated presence therapy), behavior management techniques, multicomponent interventions, and other therapies (education of patients and caregivers, exercise, and animal-assisted therapy involving real or robotic animals) (Abraha et al., 2017). Non-pharmacological strategies were even found to be more effective than pharmacological treatments (Yury and Fisher, 2007; Brodaty and Arasaratnam, 2012; Schneider et al., 2006a) and appear to have fewer adverse effects than pharmacotherapy with antipsychotics (Dyer et al., 2018). Moreover, a meta-analysis found such interventions to prove useful, versatile, and potentially cost-effective in improving outcomes and quality of life in individuals with dementia and their carers (Olazarán et al., 2010).

However, in practice, the widespread use of nonpharmacological strategies is beset by many difficulties, the main ones being lack of trained personnel, limited knowledge on the efficacy of nonpharmacological interventions, staff opinions and preferences, and an expectation of quick resolution of symptoms (Ervin et al., 2014; Jennings et al., 2018). In addition, in the case of severe agitation, or other emergency situations where patients may be endangering themselves or others, pharmacological intervention has priority. In typical situations, current evidence suggests that nonpharmacological techniques should be used as a first-line option for NPS and that many clinical guidelines recommend starting with such management (Dyer et al., 2018).

This part of the review will focus on recent advancements in non-pharmacological management (NPM) and discuss other approaches a reader can find in previous works (see Meyer and O’Keefe, 2018).

As NPM supports mainstream therapy in alleviating symptoms and improving the quality of life of patients, both approaches should be considered as parts of a broader concept of person-centered care (PPC). PPC, by supporting the personhood of patients and understanding their experience, attempts to assist them and their family in reaching the best possible quality of life (Molony et al., 2018).

The well-being of a patient with dementia can be achieved through understanding the individual needs and history of each patient. Similarly, to successfully reduce its occurrence, the underlying cause of the symptom also has to be understood. NPS can stem either from neurocognitive impairment associated with dementia or from the unmet needs of the patient; therefore, the possible interplay between these factors should be addressed while planning intervention.

In cases of dementia where cognitive functions are impaired, patients lose their ability to communicate through language. In such cases, they can sill manifest their needs, such as pain, hunger, boredom, insecurity, or anxiety, through their behavior. However, such behavior may be influenced by their cognitive impairment, which affects the way they perceive, interpret, and react to the environment: patients may have problems with explicit memory, recognition of visual motion or spatial orientation, and topographic disorientation (Zwijsen et al., 2016). A greater understanding of the mechanisms underpinning behavior can serve as a basis for more precise interventions.

Non-pharmacological interventions can be divided into direct ones targeted at patients and indirect ones targeted at their environment (Caspar et al., 2018).

Direct interventions attempt to address the needs of the patient and can include lifestyle modification (e.g., diet, activity programs) or psychological therapy (art therapy, humor, animal, or PARO robotic pets assisted therapy); in contrast, indirect interventions involve either modification of the physical environment such as room temperature, light and noise levels, or familiarity or educating caregivers and reducing the stress and burden placed on them (Marriott et al., 2000; Soril et al., 2014).

One of the most distressing symptoms for caregivers is agitation, or aggression, which also is one of the most common reasons for antipsychotic prescription. However, antipsychotic use is associated with a high risk of adverse effects (Farlow and Shamliyan, 2017), and there is an urgent need to define efficient strategies to reduce their occurrence.

Between 45 and 80% of patients with dementia in nursing homes suffer from chronic pain. Impaired cognitive functions limit the possibility of communicating pain and thereby increase the risk of this pain of being underdiagnosed and untreated (Hadjistavropoulos et al., 2007). Similarly, lack of activity concerns up to 90% of patients with dementia residing in nursing homes: A problem that has been consequently raised by researchers since 1995 (Nolan et al., 1995; Hancock et al., 2006). Pain and boredom are important determinants of agitation (Kolanowski et al., 2017), and interventions directing them have been explored as a potential way to lower the level of agitation. In one study, nursing home residents were visited by a pair of elder-clowns for approximately 10 min, twice a week, for 12 weeks. The clowns interacted with the patients using improvisation, humor, empathy, and expressive modalities such as songs, musical instruments, and dance. At the end of the intervention, the total NPI-NH scores were found to have declined significantly, and a general reduction of agitation was observed (Kontos et al., 2016).

In another study, a group of patients treated with 8 weeks of pain management demonstrated significantly lower agitation and aggression in comparison with controls (Husebo et al., 2011; Husebo et al., 2014).

PARO robotic pets, in use since 2003, have proven successful in lowering stress, depression, and anxiety in patients with dementia. Intervention with a robotic pet three times weekly per 20 min was found to reduce the need for pain medications and psychoactive medication (Petersen et al., 2017).

No significant improvement in total NPI score was observed after 3 months of supplementation with nutraceutical formulation (NF) [folate, alpha-tocopherol, B12, S-adenosyl methionine (SAM) N-acetyl cysteine (NAC), and acetyl-L-carnitine (ALCAR)] as part of the Phase II Randomized Clinical Trial of a Nutritional Formulation for Cognition and Mood in Alzheimer’s Disease (Remington et al., 2015).

Another group of interventions are those that directly focus on supporting the identity of the patients with dementia. In 2011, Caddell and Clare reviewed existing interventions and identified 10 studies aimed at promoting the preservation of patient identity. Although all interventions reported benefits to the patients, the considerable heterogeneity of patients and methodologies used in the study do not allow any firm conclusions to be drawn (Caddell and Clare, 2011). A promising area of research attempts to enhance and enrich the strategies used by patients to cope with cognitive impairment to promote well-being in the early stages of dementia. The spectrum of responses to problems with memory has been proposed to range from ‘self-maintaining’ to ‘self-adjusting’ (Clare, 2003). While patients following a self-maintaining strategy attempt to maintain the prediagnosis concept of self, those with a self-adjusting stance adapt a self-concept based on the challenges associated with the memory decline. A Preserving Identity and Planning for Advance Care (PIPAC) intervention combines these two components. The self-maintaining (reminiscence-based) component includes documenting an identity-salient role from the life history of the patient with the aim of describing what it meant to patients to “live well” in the past. The self-adjusting component is incorporated into a discussion of advance care planning (ACP), in which the patients focus on what it means for them to ‘live well’ in the future. They are informed about treatment options and how care decisions are made and rehearse communicating their preferences to relatives. After intervention, authors observed lower depressive symptoms and illness burden and greater quality of life and health-related indicators of well-being (Hilgeman et al., 2014).

Studies suggest that a combination of both direct and indirect non-pharmacological interventions may be essential in order to alleviate BPSD. Moreover, a recent study on health-related quality of life (HRQL) for people with dementia found antipsychotic discontinuation to have a detrimental effect on HRQL. This negative impact was, however, mitigated by social interactions (Ballard et al., 2017).

For intervention to be successful, it should consider the importance of a caring environment, i.e., the physical, built one, and the social environment, as well as care skill development and maintenance, and taking an individualized approach to each patient (Caspar et al., 2018).

### Pharmacological Treatments

Currently, non-pharmacological and pharmacological management options exist for treating NPSs. Of the two, pharmacological intervention seems to be the ideal solution, mainly from the perspective of the caregiver. Oral dosage form administration is quick and easier than non-pharmacological interventions; in addition, it does not require professional staff involvement and allows the strength of the effect to be changed by increasing the dose of the drug. Moreover, in real life, some caregivers are interested in the possibility of administering the drug in the form of a solution without informing the patient.

Pharmacological interventions have many limitations in specific populations such as elderly patients with dementia and behavioral symptoms.

A key problem is the relatively small number of randomised clinical trials, most of which have been carried out on a narrowly defined indication, such as apathy, depression, or anxiety: some publications employ general terms such as *psychiatric symptoms*, while others use very detailed ones such as *apathy* in patients with Huntington’s disease. Neuropsychiatric symptoms, on the other hand, occur in the majority of types of dementia, not just Alzheimer’s disease (AD). In addition to AD, studies on DLB, PDD and frontotemporal lobe degeneration have also been performed; however, many of the observations on the efficacy of psychotropics in behavioral changes are based on studies conducted in other age groups with different clinical charactristics. Most research focuses on the effectiveness of antipsychotics, mood stabilizers and antidepressants in treating NPSs, and cholinesterase inhibitors and memantine in the case of AD.

Only few drugs are indicated for treating NPSs in dementia. Tiapride is recommended for agitation in cases with dementia in Poland, and pimavanserin for psychosis associated with Parkinson’s disease in the USA. In addition, risperidone is recommended for treating persistent aggression in moderate-to-severe cases of AD not responding to non-pharmacological interventions and when there is a risk of harm to the patient; however, treatment should be restricted to 6 weeks (Canada and Europe). Finally, quetiapine is recommended for psychiatric symptoms in patients with dementia; this indication is covered by public insurance in Poland (Lanctôt et al., 2017). Although prescribing psychotropic medications to a patient with dementia appears to be clinically justified, it still remains an off-label order in most countries.

Secondly, a minority of interventions with psychotropic drugs for NPSs in nursing homes is fully appropriate (van der Spek et al., 2016). A difference between correctness of use of antidepressants (used mostly appropriately) and anticonvulsants (used mostly inappropriately) was noticed. Unfortunately, for many (frequently unclear) reasons, the main method of pharmacological treatment of NPSs is based on antipsychotics. Antipsychotics are disproportionately often used in older populations (Colenda et al., 2002; Nijk et al., 2009; Gulla et al., 2016; Maust et al., 2017) for various indications, not only psychosis. Many physicians believe that antipsychotics are multipotential: they may also be effective in other clinical conditions, and their primary activity does not conern psychoses. Therefore, their prescription is reasonable in patients with delusions, hallucinations, or psychotic anxiety (Sultzer et al., 2008). Even if prescribing a neuroleptic to a person with dementia appears to be clinically justified, it still remains an off-label order in most countries (Maglione et al., 2011). In many cases, neuroleptics act mainly *via* a non-specific sedative effect and serve as a form of chemical restraint: efficacy data indicates that for all atypical antipsychotics show at best modest benefit against neuropsychiatric symptoms observed in cases with dementia (Seitz et al., 2013).

Thirdly, antipsychotics have been consistently associated with serious adverse effects and increased mortality in patients with dementia (Schneider et al., 2005; Ma et al., 2014; Schneider et al., 2006a; Kales et al., 2012; Ralph and Espinet, 2018), with the risk being dose-dependent (Maust et al., 2015). Increased mortality is related to a range of interacting factors, and the precise mechanisms of death are still uncertain. Antipsychotic treatment can result in cerebrovascular events (e.g., stroke), cardiovascular effects (e.g., orthostatic hypotension, cardiac arrhythmias, and QTc prolongation), metabolic effects, extrapyramidal symptoms and falls, as well as pneumonia (Steinberg and Lyketsos, 2012).

The growing body of evidence regarding the increased risk related to antipsychotic use among patients with dementia resulted in black box warnings being issued by the FDA for atypical drugs (in 2005) and conventional drugs (in 2008), and as experts’ recommendations (Herrmann et al., 2013; Ihl et al., 2015). However, these guidelines have had little impact on prescribing psychotropics in some practices (Desai et al., 2012; Craig et al., 2016) and with positive trends in others, such as Danish residents aged ≥65 years (Nørgaard et al., 2016). The drug may often be prescribed in response to the request of a care giver, family member, or member of staff.

Finally, overuse of antipsychotics has been reported (Rios et al., 2017), and some measures for limiting such practices have been undertaken (Jessop et al., 2017; Kirkham et al., 2017).

However, while long-term antipsychotic treatment is known to be associated with an increased risk of mortality, their use may be justified by circumstances. Even if the decision to implement the treatment may be clinically justified, regular attempts to withdraw these drugs are recommended in guidelines (Azermai et al., 2012), and practical algorithms have been proposed for process of drug discontinuation (Miarons et al., 2017). Even so, withdrawal of these drugs has consequences, especially after long-term use, including the obvious risk of re-aggravation of NPSs. A meta-analysis published by Declercq et al. indicates that AD patients can be withdrawn from chronic antipsychotic medication without demonstrating detrimental effects on their behavior (Declercq et al., 2013); however, the precise effects of withdrawal on patient cognition, adverse events, quality of life, and decrease in mortality remain unknown (Van Leeuwen et al., 2018).

### Agitation

Agitation is quite a common problem in patients with Alzheimer-type dementia but may also occur in other types of dementing illnesses. Although non-pharmacological treatments represent first-line options, they are often of limited efficacy. This fact may explain why various categories of psychotropic drugs are used for treatment of agitation in dementia. These include typical (promazine) and atypical antipsychotics, antidepressants, anticonvulsants, antihistaminergic drugs (hydroxyzine), and herbal preparations. Most of these are off-label psychotropic medications, because there is insufficient or no data for their efficacy and safety in patients with dementia, and their prescription is based on tradition and personal opinions of physicians. Most worryingly, their use may entail serious adverse effects (SAEs). For example, a recent Cochrane meta-analysis (Baillon et al., 2018) suggests that valproate preparations, which are widely used for “organic brain disorders”, may well be ineffective at treating agitation in people with dementia. The treatment has a high rate of adverse effects, associated with possible SAEs, and hence valproate cannot be recommended for management of agitation in dementia.

Few papers have been published on the efficacy of antidepressants in agitation and psychosis in patients with dementia. A Cochrane meta-analysis concluded that the citalopram and sertraline were more effective in reducing symptoms of agitation compared to placebo in two studies (Seitz et al., 2011). SSRIs and trazodone were also found to be well tolerated when compared to typical and atypical antipsychotics. In addition, no differences were observed between antidepressants and typical or atypical antipsychotics in terms of efficacy.

A recent systematic review and meta-analysis of RCTs performed to determine the most efficacious and acceptable treatments of agitation in dementia found that haloperidol demonstrated little efficacy compared to placebo, despite its relatively widespread use for alleviating agitation (Kongpakwattana et al., 2018). In addition, dextromethorphan/quinidine and risperidone were significantly more efficacious than placebo, as were SSRIs when considered as a class, but not when analyzed individually.

Moreover, some completed randomized controlled trials (RCTs) on treating agitation in dementia of Alzheimer-type with new or repositioned drugs have been published recently (Porsteinsson and Antonsdottir, 2017). Considering the available data on drug efficacy, adverse effects, availability, and novel drug registration procedures, it seems that citalopram may be the most sensible option for many physicians in controlling agitation in AD (Porsteinsson et al., 2014). However, the treatment period should be at least nine weeks long to allow enough time for full response (Weintraub et al., 2015). An alternate algorithm of drug treatment for agitation and aggression associated with AD or mixed dementia was proposed by Davies et al. (2018). The authors recommend starting treatment with risperidone, then aripiprazole or quetiapine, followed by carbamazepine and then citalopram. In the case of citalopram prescription, it is important to be aware of the increased risk of QTc prolongation, which can be problematic in geriatric patients. Promising novel and/or repositioned drugs intended for agitation in dementia are characterized in **Tables 1** and **2**.

**Table 1:** Current studies on pharmacological treatment of agitation and psychosis in dementia with novel drugs [data available at: ClinicalTrials.gov (accessed June 30, 2020); filters used: agitation, psychosis, and dementia; Studies: recruiting; not yet recruiting; active, not recruiting; enrolling by invitation]; PLC, placebo; PD, Parkinson’s disease; AD, Alzheimer’s disease; FTD, fronto-temporal dementia.

Name	Mechanism of action	Study population	Treatment	Results/status of the study	Study ID (ClinicalTrial.gov) or reference
Pimavanserin	A selective 5-hydroxytryptamine (HT)2A receptor inverse agonist/antagonist	PD psychosis	Pimavanserin 34 mg vs. PLC	Significant improvement with pimavanserin vs. PLC (-5·79 decrease in SAPS-PD scores in pimavanserin group compared with -2·73 for PLC (difference -3·06, 95% CI -4·91 to -1·20; p=0·001; Cohen’s d 0·50))	ACP-103-020; (Cummings et al., 2014)
		AD psychosis	Pimavanserin 34 mg vs. PLC	Significant improvement for pimavanserin Primary endpoint (week 6): Mean change in the Neuropsychiatric Inventory-Nursing Home version psychosis score Pimavanserin versus PLC: -3·76 points (SE 0·65) versus -1·93 points (0·63) (mean difference -1·84 [95% CI -3·64 to -0·04], Cohen’s d=-0·32; p=0·045); No significant advantage for pimavanserin versus PLC at week 12 (treatment difference -0·51 [95% CI -2·23 to 1·21]; p=0·561);	ACP-103-019; (Ballard et al., 2018)
		AD psychosis	Pimavanserin 34 mg vs. PLC	Significant efficacy in patients with higher baseline severity of psychotic symptoms (delta=-4.43, Cohen’s d=-0.73, p=0.011); Pimavanserin vs PLC: ≥30% improvement was 88.9% vs. 43.3% (p<0.001); ≥50% improvement was 77.8% vs. 43.3% (p=0.008);	ACP-103-019; (Ballard et al., 2019)
		Dementia-related psychosis	Pimavanserin (34 mg and 20 mg) vs. PLC	No Study Results Posted on ClinicalTrials.gov for this Study; Study has been completed, results have not been published;	NCT03325556; [ACP-103-045]; 2017-002227-13 (EudraCT Number)
		PD psychosis	A retrospective chart review	Clinical improvement in psychosis documented in 76% of patients (69/91)	(Sellers et al., 2019)
Scyllo-inositol (ELND005)	Iinhibition of amyloid beta peptide aggregation	Agitation and aggression in AD	A prospective, 12-week, Randomized, Double-Blind, Placebo-Controlled, Phase 2 Efficacy and Safety Study of Oral ELND005 for Treatment of Agitation and Aggression in Patients With Moderate to Severe AD	Study has been completed, results have not been published; Study Results have been posted on ClinicalTrials.gov;	NCT01735630; ELND005-AG201
		agitation and aggression in AD	36-week extension study of Study AG201	Study has been terminated, results have not been published; Study Results have been posted on ClinicalTrials.gov;	NCT01766336
Mibampator (LY-451395)	An amino-3-hydroxy-5-methyl-4-isoxazole propionic acid receptor potentiator	Agitation and aggression in AD	3 mg of mibampator orally twice daily for 12 weeks (may have been reduced to 1 mg if participant was unable to tolerate) vs PLC	No significant group differences; mibampator was significantly better (p = 0.007) than PLC only on the Frontal Systems Behavior Inventory	NCT00843518; (Trzepacz et al., 2013)
Lumateperone (ITI-007)	A potent 5-HT2A antagonist, a mesolimbic/mesocortical dopamine phosphoprotein modulator (DPPM) with pre-synaptic partial agonist and post-synaptic antagonist activity at D2, a glutamate GluN2B receptor phosphoprotein modulator with D1-dependent enhancement of both NMDA and AMPA currents *via* the mTOR protein pathway and an inhibitor of serotonin reuptake	Agitation in patients with dementia, wncluding Alzheimer’s disease	A phase 3, 4 week, Randomized, Double-Blind, Placebo-Controlled, Multi-Center Study (ITI-007 9mg/d vs PLC)	Study terminated; no data published	NCT02817906; (Kumar et al., 2018)
ORM-12741	A potent and selective alpha-2C adrenoceptor (AR)-antagonist	Agitation/Aggression Symptoms in AD	A randomised, 12-week, Double-blind, Placebo-controlled, Parallel Group, Multicentre Study; ORM-12741 low dose twice a day and ORM-12741 high dose twice a day vs PLC	No study results posted on ClinicalTrials.gov for this Study; study has been completed, results have not been published;	NCT02471196
MP-101 (other names: LY2979165, LY2812223)	LY2979165 is the alanine prodrug of LY2812223, a selective and potent orthosteric mGlu2 receptor agonist	Dementia-related psychosis and/or agitation and aggression	A phase 2, 10-week, double blind, placebo controlled study of MP-101 (escalating dose of MP-101 administered orally once daily, starting at 20 milligrams up to 60 milligrams vs PLC)	Study has been terminated, results have not been published (statistical futility; totality of evidence suggests study unlikely to meet endpoint); study results have not been posted on ClinicalTrials.gov;	NCT03044249

**Table 2:** Current studies on pharmacological treatment of agitation and psychosis in dementia with repositioned drugs [data available at: ClinicalTrials.gov (accessed June 30, 2020); filters used: agitation, psychosis, and dementia; Studies: recruiting; not yet recruiting; active, not recruiting; enrolling by invitation]; PLC, placebo; PD, Parkinson’s disease; AD, Alzheimer’s disease; FTD, frontotemporal dementia.

Name	Mechanism of action	Study population	Treatment	Results/status of the study	Study ID (ClinicalTrial.gov) or reference
Deuterated (d6)-dextromethorphan/quinidine (AVP-786)	Dextromethorphan - a low-affinity N-methyl-D-aspartate receptor antagonist, σ1 receptor agonist, serotonin and norepinephrine reuptake inhibitor, and neuronal nicotinic α3β4 receptor antagonist; Quinidine - an anti-arrhythmic agent blocking voltage-gated sodium channels, inhibitor of cytochrome P450 2D6; Quinidine increases the bioavailability of dextromethorphan and prolongs its effects	Agitation in patients with Alzheimer’s type dementia	A phase 3, 12-week, multicenter, randomized, double-blind, placebo-controlled, parallel-design Study (AVP-786 (Dose 1) vs AVP-786 (Dose 1) Vs PLC)	Ongoing (study is recruiting participants)	NCT03393520
			
		Agitation in patients with Alzheimer’s type dementia	A phase 3, multicenter, long-term, extension study for 56 weeks	Ongoing (study is recruiting participants)	NCT02446132
		Agitation in patients with Alzheimer’s type dementia	A phase 3, 12-week, multicenter, randomized, double-blind, placebo-controlled Study	Study has been completed, results have not been published;	NCT02442778
Brexpiprazole (OPC-34712)	Second generation antipsychotic	Agitation associated with Alzheimer’s type dementia	A phase 3, 12-week, multicenter, randomized, double-blind, placebo-controlled, fixed-dose trial (three arms: Low Dose Brexpiprazole Arm vs. High Dose Brexpiprazole Arm vs PLC)	Ongoing (study is recruiting participants)	NCT03548584
		Agitation associated with Alzheimer’s type dementia	A phase 2/3 multicenter, placebo-controlled, randomized, double-blind, parallel-group Comparison Trial (brexpiprazole 1 mg vs. brexpiprazole 2 mg vs. PLC for 10-week treatment regimen)	Ongoing (study is recruiting participants)	NCT03620981
		The long-term safety and tolerability of oral brexpiprazole in agitation associated with Alzheimer’s type dementia	A 12-week, multicenter, active-treatment extension trial to evaluate the safety and tolerability of brexpiprazole; brexpiprazole oral tablet 2 mg/day vs. Oral tablet 3 mg/day; taken once daily.	Ongoing (study is recruiting participants)	NCT03594123
		Agitation associated with Alzheimer’s type dementia	A multicenter, oncontrolled, open-label trial of brexiprazole 1 mg or 2 mg for a 14 week treatment regimen	Ongoing (study is recruiting participants)	NCT03724942
Prazosin	Postsynaptic alpha-1 adrenoreceptor antagonist	Disruptive agitation in AD	Oral prazosin hydrochloride capsules will be administered twice daily, with individualized doses up to a maximum of 4 mg mid-morning and 6 mg at bedtime, or matching PLC capsules	Ongoing (active, not recruiting)	NCT03710642
		Agitation/aggression in AD	Prazosin (drug was initiated at 1 mg/day and increased up to 6 mg/day using a flexible dosing algorithm) vs. PLC for 8 weeks	Prazosin was well tolerated and improved behavioral symptoms in patients with agitation/aggression in AD	NCT00161473; (Wang et al., 2009)
		Disruptive agitation in AD	Prazosin: 4 mg capsules twice daily for 12 weeks vs. PLC capsules twice daily for 12 weeks	Study has been completed, results have not been published; study results have been posted on ClinicalTrials.gov;	NCT01126099
Cannabinoids	Ligands of cannabinoid receptors CB1 and CB2	Dementia related agitation and aggression	A phase 2, Randomized, Double-blind, Placebo-controlled Trial (cannabis oil containing Δ9-Tetra-Hydrocannabinol (Δ9-THC) and Cannabidiol (CBD) in a 1:20 ratio and at a concentration of 30% CBD and 1.5% Δ9-THC. Each oil drop contains about 12 mg CBD and 0.6 mg Δ9-THC)	Ongoing (study is recruiting participants)	NCT03328676
Mirtazapine	Noradrenergic and specific serotonergic tetracyclic antidepressant (NaSSA)	Agitation in dementia	A Pragmatic, Multi Centre, 12-week, Double-blind, Placebo Controlled Randomised Trial of mirtazapine vs. PLC. Participants will then be followed up for 1 year after	Ongoing (active, not recruiting)	NCT03031184
Lithium	Mood stabilizer; exact mechanism of action in mood regulation has not been clarified; inhibitor of glycogen synthase kinase 3β (GSK3)	Behavioral symptoms in FTD	A phase 2, 12-week, double blind, placebo controlled study of Lithium (escalating dose of lithium, starting at 150 mg/day, with subsequent dose titration to 300, 450, and 600 mg/day as tolerated according to side effects and blood lithium level)	Ongoing (study is recruiting participants)	NCT02862210
		Psychosis and agitation in AD	A phase 2, randomized, double-blind, placebo-controlled, 12-week trial of lithium (escalating dose of lithium, starting at 150mg/day, with subsequent dose titration to 300mg/day at the 2-week visit, 450mg/day at the 4-week visit, and 600mg/day (maximum daily dose) if tolerated and based on real lithium blood level)	Study has been completed, results have not been published; study results have been posted on ClinicalTrials.gov;	NCT02129348
Escitalopram	Selective serotonin reuptake inhibitor	Agitation in AD	a phase 3, randomized, double-blind, placebo-controlled, 12-week trial (5-15 mg/day (target: 15mg/day if tolerated) of escitalopram vs. PLC)	Ongoing (study is recruiting participants)	NCT03108846; S-CitAD

An alternative method of treating agitation in dementia is by electroconvulsive therapy (ECT). A recent review of papers investigating the use of ECT for treating agitation in dementia (Glass et al., 2017) identified 11 papers, with a total number of 216 patients. The studies indicate promising results in decreasing agitation in patients with dementia; however, the studies have many methodological limitations regarding the type of study, use of psychotropic medications, choice of scales, lack of control group and numer of patients, among others.

### Psychotic Features

Most psychosis symptoms that occur in dementia are hallucinations and delusions, and many patients require antipsychotic treatment to deal with of such distressing psychiatric symptoms. This is especially true when a patient acts on the delusions, experiences significant fear, or if their safety is threatened.

Antipsychotics are still widely prescribed, even in cases of dementia without psychosis. While a decline of first generation antipsychotic drug prescriptions was observed following a UK National Guidance and Drug Safety Warning, by the National Institute for Health and Care Excellence, the decreasing trend in second-generation drug prescriptions has been halted by the increased prescription of risperidone (Stocks et al., 2017).

In 2016, the American Psychiatric Association published a set of Practice Guidelines on the use of antipsychotics to treat agitation or psychosis in dementia (Reus et al., 2016). The guidelines comprise 15 statements on antipsychotic use in dementia, grouped into five sections: assessment of behavioral/psychological symptoms of dementia; development of a comprehensive treatment plan; assessment of benefits and risks of antipsychotic treatment for the patient; dosing, duration, and monitoring of antipsychotic treatment; and use of specific antipsychotic medications depending on clinical context. Although Reus et al. (2016) indicate that “guidelines should not be considered as a statement of the standard of care or inclusive of all proper treatments or methods of care”, such guidance regarding the method of assessing the need for antipsychotic treatment and monitoring results may nevertheless be of value to clinicians.

The efficacy and safety of the antipsychotics olanzapine, quetiapine, and risperidone in treating dementia were examined in the CATIE-AD study (Ismail et al., 2007; Sultzer et al., 2008; Schneider et al., 2006b). Other second-generation antipsychotics, such as aripiprazole and ziprasidone, have also demonstrated safety and efficacy in treating AD (De Deyn et al., 2005; Rocha et al., 2006; Mintzer et al., 2007; Streim et al., 2008), as well as in dementia with Lewy Bodies (Lee and Shen, 2017; Sugawara Kikuchi and Shimizu, 2019), where neuroleptic treatment is problematic due to neuroleptic hypersensitivity.

Currently, more data is needed to conclusively determine whether different atypical antipsychotics vary with regard to their effectiveness, or their risk of mortality or cerebrovascular events (Yunusa et al., 2019). Novel drugs such as pimavanserin in synucleinopathies and brexiprazole are undergoing evaluation in various populations of patients with dementia (**Table 1**).

### Apathy

Apathy is a non-cognitive symptom and one of the most prevalent behavioral and psychological symptoms of dementia, which can be observed even at the prodromal stage (Sherman et al., 2018). It can be characterized as diminished motivation or even lack of motivation and loss of initiative. Apathy is a longlasting state that is associated with increased mortality and a substantially greater burden for caregivers (Harrison et al., 2016; Camargo et al., 2016; Nijsten et al., 2017; Terum et al., 2017); however, a Japanese study found apathy, anxiety, and depression not to seriously aggravate caregiver burnout. A higher level of burnout was related to agitation/aggression, irritability, aberrant motor behavior, and hallucinations (Hiyoshi-Taniguchi et al., 2018).

Although our understanding of the underlying neuronal basis of apathy has improved in recent years, the effectiveness of treatment is still limited (Huey et al., 2017; Lansdall et al., 2017; Ducharme et al., 2018; Fernández-Matarrubia et al., 2018; Kumfor et al., 2018). The treatment of apathy includes both non-pharmacological and pharmacological strategies and varies according to the type of dementia. A number of non-pharmacological interventions have been employed: music-based interventions, regular individualized one-on-one personal contact, the use of cognitive stimulation therapy, multi-sensory behavior therapy, behavioral and environmental interventions, group art therapy, the use of therapeutic conversation, reminiscent group therapy, and Snoezelen-based care (Goris et al., 2016). However, a review of existing evidence shows it to be an underexplored field (Theleritis et al., 2018).

Cholinesterase inhibitors, memantine, antidepressants, antipsychotics, psychostimulants, and drugs with various mechanisms of action have demonstrated mixed results or no efficacy at all for treating apathy in Alzheimer’s disease (Sepehry et al., 2017; Ruthirakuhan et al., 2018). Agomelatine, but not paroxetine, was associated with a significant reduction of apathy in FTD (Deakin et al., 2004; Callegari et al., 2016), while bupropion was ineffective in the treatment of apathy in Huntington’s disease (Gelderblom et al., 2017). Apathy is a substantial part of clinical picture of Parkinson’s disease (den Brok et al., 2015), Parkinson’s disease dementia, and dementia with Lewy bodies, significantly affecting the course of the disease in the case of the latter (Breitve et al., 2018). However, no efficatious treatment currently is known to exist (Santangelo et al., 2013; Holguín Lew et al., 2017).

### Depression

Depression is inextricably linked to cognitive disorders and dementia. Over the years, there has been a discussion about the relationship between depression and dementia (Bennett and Thomas, 2014). On the one hand, it was postulated that depression is a risk factor or a causative factor of dementia. On the other hand, depression has been proven to be a typical presentation of the initial phases of dementia or MCI and is in fact part of the clinical picture of dementia. There is also a proposal that antidepressant treatment is responsible for the occurrence of dementia (Lee et al., 2016; Moraros et al., 2017; Wang C. et al., 2018), especially when inappropriate medication is ised (Heser et al., 2018). Regardless of neuropathological and pathophysiological conditions, depression during dementia is a significant clinical and therapeutic problem with serious consequences for the patients and caregivers.

As with all BPSD, the management of clinical depression should start with the optimization of dementia treatment. Unfortunately, while acetylcholinesterase inhibitors and memantine are effective in the symptomatic treatment of AD, current evidence suggests that they have limited efficacy for the treatment of depressive symptoms in dementia. Furthermore, non-pharmacological treatments, which are a preferred initial approach for all NPSs, have limited evidence for depressive symptoms. However, a recent review identified five modifiable relevant factors related to depression in dementia among community-dwelling individuals: pain, neuropsychiatric symptoms, cognitive decline, social isolation, and quality of life; in addition, neuropsychiatric symptoms and quality of life were found to be modifiable factors for patients living in long-term care facilities (Kubo et al., 2019). The authors conclude that non-pharmacological interventions improving identified relevant factors may improve symptoms of depression in patients with dementia. Moreover, symptoms of depression and anxiety can be reduced by psychological interventions added to usual care (Orgeta et al., 2014; Orgeta et al., 2015).

While planning to implement non-pharmacological interventions for depression in patients with dementia, it should be considered that the presence of depression may be an important barrier to engagement in therapy, for example, physical activity (Watts et al., 2018).

In the majority of patients, however, pharmacological treatment is the basis of therapy.

A practical question arises whether there is sufficient evidence for recommending the use of pharmacotherapy in treating depression in patients with dementia (Farina et al., 2017; Ford and Almeida, 2017). Serotonergic drugs are a basic option in the treatment of mood disorders in the general population. It seems that they are also a good option for mood disorders in people with cognitive impairment and dementia (Magierski and Sobow, 2016). However, a recent Cochrane meta-analysis found little support for the efficacy of antidepressants for treating depression in dementia (Dudas et al., 2018).

### Sleep Problems

Sleep disorders in patients with dementia are frequent, affecting between 25 and 80% of patients; these figures are higher than those associated with healthy aging and are believed to result from neurodegenerative processes (Ohayon et al., 2004; Bombois et al., 2010). The consequences of abnormal sleep in general population are increased risk of cognitive impairment and dementia (Bubu et al., 2017; Shi et al., 2018), but in patients with dementia, consequences include comorbidity, risk of falling, poorer quality of life, and increased psychological, physical, and financial burdens in the caregiver. In addition, sleep disorders are the primary risk factor for nursing home placement, even more so than cognitive impairment. Finally, they often aggravate the course of dementia through drowsiness during the day, thus impairing cognitive performance, driving, and social activities (Tractenberg et al., 2005).

Different sleep disorders are observed depending on the type of dementia (Roth, 2012). Alzheimer’s disease is characterized by an irregular sleep-wake rhythm, sundowning, wandering, and obstructive sleep apnea. PDD is characterized by REM sleep behavior disorder, sleep maintance insomnia, hypersomnia, restless leg syndrome/periodic limb movements in sleep, while DLB patients demonstrate REM sleep behavior disorder, hypersomnia, periodic limb movements in sleep, and irregular sleep-wake rhythms. Similarly, those observed in FTDs include insomnia, excessive daytime sleepiness, sleep disordered breathing, and less frequent restless leg syndrome (McCarter et al., 2016).

It is difficult, or even impossible, to propose a universal method of treating sleep disorders in dementia due to this significant variation in clinical picture and neuropathology. Popular drugs for sleep disturbances in dementia include melatonin, trazodone, benzodiazepines, Z-drugs (zolpidem, zopiclone, and zaleplon), and recently registred ramelteon. At this point, it is necessary to recall that elderly benzodiazepine users became more sensitive to their medications. Paradoxical excitement (increased anxiety, acute excitement, and hyperactivity) can be observed in some cases. Moreover, the Z-drugs have documented night time unrecalled events, which may endanger other residents, and ramelteon may have a delayed onset for therapeutic effects of days. Other treatment modlaities include antihistaminergic drugs, herbal preaprations, or antidepresants (for example, mianserine and mirtazapine). A Cochrane meta-analysis examining the efficacy of pharmacotherapies for sleep disturbances in dementia found a lack of evidence regarding the issue of sleep problems in dementia (McCleery et al., 2016).

## Network Meta-Analyses for Pharmacological and Non-Pharmacological Treatments of BPSD

A sizable numer of papers have been published on the effectiveness of different strategies targeting BPSD. As a result, both pharmacological and non-pharmacological methods are recommended through guidelines. Due to the diversity and extensiveness of interventions (exercise versus reminiscence therapy versus antipsychotic use) and the lack of head-to-head trials, it is difficult or even impossible to synthesize and objectify present data. This applies especially to descriptive literature reviews. Network meta-analysis solves this problem, because it allows findings to be analyzed quantitatively and for direct and indirect evidence to be evaluated simultaneously.

Several systematic reviews and network meta-analyses on the efficacy of different strategies for BPSD treatment were published recently. These publications examine the effectiveness of various therapeutic options and make head-to-head comparisons of the effectiveness of the tested drugs (Kongpakwattana et al., 2018).

A comparison of pharmacological and nonpharmacological interventions for treating aggression and agitation in adults with dementia (Watt et al., 2019) found showed that multidisciplinary care, massage and touch therapy, and music combined with massage and touch therapy were clinically more efficacious than usual care. Despite the study limitations, including high risk of bias related to outcome missing data, it was found that nonpharmacological interventions seemed to be more efficacious than pharmacological interventions for reducing aggression and agitation in adults with dementia. However, the study did not evaluate the harm and costs of the analyzed therapies. The effectiveness of non-pharmacological methods in managing of agitation in patients with dementia has also been confirmed elsewhere (Leng et al., 2020).

Another assessment of the comparative efficacy and safety of pharmacological and non-pharmacological therapies for treating BPSD (Jin and Liu, 2019) based on data from 146 randomized trials comprising 44,873 patients found that the antipsychotics aripiprazole, haloperidol, quetiapine, and risperidone demonstrated significant efficacy compared to placebo, while memantine, galantine, and donepezil have had the least. Importantly, all drugs were found to demonstrate acceptable safety, and the authors conclude that drug therapy should be the first option in the treatment of BPSD.

Similar results were obtained from a Bayesian network meta-analysis on the efficacy of cholinesterase inhibitors in patients with mild-to-moderate AD by Kobayashi et al. (2016) who conclude that ChEIs should have significant efficacy for cognition and global change assessment, but the efficacy on BPSD is questionable.

## Promising Therapeutic Options of BPSD

As current strategies for the management of NPSs often lack effectifveness, there is a need to identify other treatment options. Although most studies focus on pharmacological interventions, some involve techniques known for their efficacy in a other clinical field. Noninvasive brain stimulation methods such as repetitive transcranial magnetic stimulation, (rTMS) and transcranial direct current stimulation (tDCS) have been tested in depression, schizophrenia, autism, and cognitive deficits in AD and MCI (Wei et al., 2017; Barahona-Corrêa et al., 2018; Cruz Gonzalez et al., 2018; Osoegawa et al., 2018). A meta-analysis of randomized controlled trials found rTMS protocols to demonstrate efficacy but not tDCS (Vacas et al., 2018); however, both were found to demonstrate safety and tolerability in the studied population.

## Summary

BPSD are a significant problem in everyday clinical practice due to the prevalence, severity of symptoms, burden on the caregiver, and difficulties in treatment. Many existing clinical guides recommend the use of non-pharmacological methods as the first course of action, and that pharmacotherapy should be used as a secondary option or when there is severe presentation of symptoms. In practice, a range of drugs are used, although most are antipsychotics. Unfortunately, many of the pharmacological options lack strong evidence from clinical trials confirming their effectiveness, and many others are used as off-label treatments.

## Author Contributions

RM and TS determined the outline. RM, ES, and DR reviewed the literature and wrote the manuscript. TS and DR reviewed and approved the manuscript.

## Funding

Swedish Research Council (Drn 2012-2291) by grants provided by the Stockholm County Council (ALF project) and CIMED. None of the sponsors had any involvement in the design of the study, the data collection or analysis, the writing of the report or the decision to submit the paper for publication.

## Conflict of Interest

The authors declare that the research was conducted in the absence of any commercial or financial relationships that could be construed as a potential conflict of interest.
